# Food vs. Fuel: Diversion of Crops Could Cause More Hunger

**DOI:** 10.1289/ehp.116-a254

**Published:** 2008-06

**Authors:** David J. Tenenbaum

Eager to promote nonpetroleum energy sources to reduce dependence on oil imports and slow global warming due to fossil fuel emissions, the United States, Brazil, and the European Union are promoting biofuels made from food crops. Ethanol production (mainly in the United States and Brazil) tripled from 4.9 billion gallons to almost 15.9 billion gallons between 2001 and 2007, according to C. Ford Runge, a professor of agricultural economics at the University of Minnesota. During that same period, biodiesel production (mainly for sale in the European Union) rose almost 10-fold, to about 2.4 billion gallons, although further expansion is now uncertain. Biofuel production has been prodded by government initiatives such as subsidies and tax incentives.

But action is not necessarily the same thing as progress, say some experts. “We are witnessing the beginning of one of the great tragedies of history,” says Lester Brown, an analyst of global resources who founded the Worldwatch Institute and now heads the Earth Policy Institute. “The United States, in a misguided effort to reduce its oil insecurity by converting grain into fuel for cars, is generating global food insecurity on a scale never seen before.”

The head of Nestlé, the world’s largest food and beverage company, agrees. As reported 23 March 2008 by Agence France-Presse, chairman and chief executive Peter Brabeck-Letmathe said, “If as predicted we look to use biofuels to satisfy twenty percent of the growing demand for oil products, there will be nothing left to eat. To grant enormous subsidies for biofuel production is morally unacceptable and irresponsible.”

Even as growing quantities of corn and other grains are being diverted for use as biofuel feedstocks, newly affluent people—mainly in Asia—are eating more meat and dairy, which puts a further demand on animal feed supplies. There are many signs of concern. On 14 April 2008, the online African Energy News Review news service noted that food riots had killed five people in Haiti, adding, “The diversion of food crops to biofuel production was a significant factor contributing to global food prices rocketing by 83% in the last year, and causing violent conflicts in Haiti and other parts of the world.”

In December 2007, the United Nations Food and Agriculture Organization (UN FAO) calculated that world food prices rose 40% in 12 months prior, and the price hikes affected all major biofuel feedstocks, including sugarcane, corn, rapeseed oil, palm oil, and soybeans. On 17 December 2007, the *International Herald Tribune* quoted FAO head Jacques Diouf warning of “a very serious risk that fewer people will be able to get food,” particularly in the developing world. In the summary proceedings of the First FAO Technical Consultation Bioenergy and Food Security, held 16–18 April 2007 in Rome, authors from a group of UN agencies cautioned that “possible income gains to producers due to higher commodity prices may be offset by negative welfare effects on consumers, as their economic access to food is compromised.” (“Welfare” here refers to standard of living, not government payments.)

“I think it is hardly in dispute anymore that the push by the U.S. and E.U. governments for a strong contribution and a mandated amount of biofuels to their energy mix has contributed to some of the food crisis problems we see today,” says Liane Schalatek, associate director of the Heinrich Böll Foundation North America, a German-based nonprofit. Indeed, policy makers have suddenly begun to reconsider the biofuel mandate in light of the global food crisis.

## A Confluence of Factors

To be fair, no one is blaming the rapid price increases solely on biofuels—hunger and malnutrition were widespread before the biofuels boom began. According to the UN World Food Programme, 854 million people were undernourished in 2001–2003, and about 10 million people die of hunger and hunger-related diseases in an average year. However, demand for biofuel feedstocks is overwhelming a food supply system that was already overextended by surging demand. Moreover, the demand for biofuel affects even nonfeedstock crops, such as rice and wheat, as farmers plant feedstocks instead of food. The price of rice hit a record 3 April 2008, according to Forbes.com Market Watch, which added that “the World Bank estimated that 33 countries faced ‘social unrest’ because of soaring food and energy prices.”

As food becomes scarce, Brown says, major exporters, including Vietnam, Russia, Argentina, and Kazakhstan, have imposed limits on exports. On 19 January 2008, *The New York Times* reported, “Egypt has banned rice exports to keep food at home, and China has put price controls on cooking oil, grain, meat, milk, and eggs.” The article added, “Just in the last week, protests have erupted in Pakistan over wheat shortages, and in Indonesia over soybean shortages . . . [and] food riots have erupted in recent months in Guinea, Mauritania, Mexico, Morocco, Senegal, Uzbekistan, and Yemen.”

High prices are also pinching food aid. According to *Rising Food Prices Intensify Food Insecurity in Developing Countries*, a February 2008 report from the U.S. Department of Agriculture (USDA) Economic Research Service, the global food aid budget would need to rise about 35% over the next decade in order to maintain the 2006 level of 8 million tons of food aid.

Meanwhile, biofuel production is booming around the world. Brazil, the United States, and Europe account for the lion’s share of today’s biofuel production and consumption. However, developers are beginning to take advantage of the many crops grown elsewhere that can be converted into fuel. In Malaysia and Indonesia, where vast palm oil plantations are being established in cleared rainforests, biodiesel refineries have created a palm oil shortage. The 19 January 2008 *New York Times* reported that the price of palm oil for cooking has risen by 70%, and street vendors in Malaysia are having difficulty finding cooking oil.

China has an active biofuels program. According to the Spanish-based nonprofit GRAIN, China has begun importing the root vegetable cassava as a feedstock from Malaysia, the Philippines, Indonesia, and Nigeria. Ironically—given that these imports will place upward pressure on the price of this dietary staple in the source countries—the GRAIN website noted that China said its motive was to “relieve tensions with food supplies.”

In Tanzania, GRAIN reports in the November 2007 white paper “An African Call for a Moratorium on Agrofuel Developments,” thousands of rice and maize farmers are being evicted from their lands in order for large companies to plant sugarcane and jatropha trees (whose seeds are a feedstock).

In *Agrofuels in Africa: The Impacts on Land, Food and Forests*, a July 2007 report from the African Biodiversity Network, Josea Doussou Bodjrenou of the Beninese nongovernmental organization Nature-Tropicale, reported that industrial groups from Malaysia and South Africa have already scouted locations in Benin for growing feed-stocks, and have proposed the conversion of 300,000–400,000 hectares in the southern Beninese wetlands for production of palm oil. Benin’s growing population will need more food, the author wrote, “but it is clear that the production of biofuels will drive farmers to allocate less land to food crops.”

In the United States, the biofuel debate largely concerns corn, the source of virtually all American fuel ethanol. By the end of 2008, says agricultural economist Jay O’Neil of Kansas State University, 18% of the U.S. corn harvest—up from 14% in 2006—could be converted to ethanol. Because the United States produces 60–70% of world corn exports, and corn is one of the largest three grain crops, U.S. corn plays a heavyweight role in the world food picture as sustenance for both humans and livestock. Corn reached a record of $6.03 per bushel in April, although prices for wheat, rice, and soybeans, the other top crops, have also recently hit records.

The ethanol boom is good news for corn farmers, and the National Corn Growers Association solidly backs growth in ethanol. Geoff Cooper, the association’s director of ethanol and business development, says there is enough corn to go around. “The mere fact that USDA expects 1.4 billion bushels of U.S. corn to be left over after all demands are met is a signal that corn availability for food and feed has not been adversely impacted,” he says. Furthermore, he says, per-acre corn production rises virtually every year: “The multiplying effect of planting more acres to corn and achieving higher yields per acre through better management practices and better technology should result in the production of enough corn to satisfy all market demands.”

Cooper also points to a factor that ethanol critics usually overlook: A by-product of ethanol production known as distillers grains can be blended into animal feed. Therefore, Cooper says, about one-third of the original feed value of the corn entering the ethanol process returns to the food supply.

## “A Perfect Storm”

O’Neil says the biggest factor in soaring world grain prices was a “perfect storm” of poor weather in the breadbasket regions during 2007, including a severe drought in Australia and poor growing conditions in Russia, Eastern Europe, and parts of the United States. “The increase in prices we have seen lately is not by any means solely related to ethanol,” he says. “It’s not even primarily related to ethanol, although ethanol does have an impact.”

Other observers see a tighter link between ethanol and food prices. For example, Runge and fellow University of Minnesota economics professor Benjamin Senauer wrote in the May/June 2007 issue of *Foreign Affairs*, “The enormous volume of corn required by the [U.S.] ethanol industry is sending shock waves through the food system. . . . By putting pressure on global supplies of edible crops, the surge in ethanol production will translate into higher prices for both processed and staple foods around the world.”

In the December 2007 issue of the International Monetary Fund publication *Finance and Development*, Simon Johnson, director of that organization’s research department, wrote, “A substantial inflationary shock in the form of higher food prices [during the previous 12 months was] driven in large part by biofuels policy in industrial countries.”

Although diversion of food crops to biofuels is a concern, John Hoddinott, an expert on economics and nutrition at the International Food Policy Research Institute (IFPRI), says some critics overstate the case. “When people describe . . . a global catastrophe, taking food from the mouths of children, they are being incautious.” Still, he says, “There are certainly vulnerable people, very poor people in very poor places—[but] there is a second group for whom the situation will be problematic but manageable.”

Livelihood is a key factor in how heavily biofuel production affects any given nation, Hoddinott adds. “If you are a net producer, a rise in the food price is good for you: you have a surplus, and you make more money. But if you are a net consumer, a rise in price is definitely not good news.” The biggest threat is in Africa, Hoddinott says. “Among very poor households, food is probably seventy to eighty percent of the budget, so proportionately, a big price change matters a lot more.”

Brown agrees that price hikes matter most to the poor, who already spend much of their income on food. “I think a lot of those on the lower rungs of the global economic ladder and barely hanging on will simply lose their grip,” he says. “The question is how many, but no one knows the answer to that.”

The ability for farmers to earn money selling biofuel feedstocks “sounds very good on the surface,” says Schalatek. “But [biofuels] can replace existing production patterns of small farmers with large-scale monoculture plantations . . . and the people who used to be farmers are turned into farm laborers.” For this reason, she says, critical civil society observers and organizations around the world prefer the term “agrofuels” over “biofuels” to reflect that these fuels are a product of corporate industrial farming, driven primarily by large international agribusinesses.

“With a focus on small-scale farming and relevance for the poor,” Schalatek asserts, “the focus would be less on biofuels but more on biomass use as an energy source for the poor.”

Biofuel production does not pose a threat of starvation in the United States, although ethanol production is helping press prices higher. Still, the soaring price of corn is having a sobering impact on the ethanol industry, which is showing signs of retrenchment after a period of phenomenal growth. In late February, grain giant Cargill cited “market conditions” (in other words, expensive corn) as it suspended plans for a 100-million-gallon-per-year ethanol plant in Kansas. And in Malaysia, a plant built to convert palm oil to 110,000 tons of biodiesel a year has yet to open, due to the high price of palm oil.

Nonetheless, the Renewable Fuels Association, a trade organization, still anticipates that the current U.S. ethanol capacity of 6.5 billion gallons per year will essentially double under current expansion plans, which call for 6.2 billion gallons of new capacity. The U.S. Energy Independence and Security Act of 2007 calls for 36 billion gallons of ethanol from corn and cellulosic feedstocks by 2022. Because cellulosic ethanol is years away from industrial production, those gallons are expected to come largely from corn, at least in the foreseeable future.

In the 2007 report *Agricultural Projections to 2016*, the USDA estimated that ethanol would absorb 31% of the U.S. corn crop in 2016. Still, Cooper contends that farmers can supply mobility and nutrition: “Our position is that there is no need to choose between using corn for feed—and food—and fuel. We can do both, and we are doing both. The emergence of the ethanol industry has not affected the availability of corn for human food and livestock feed uses. In fact, more U.S. corn is being used for feed and human food use in 2008 than was used for those purposes in 2007.” However, *Ethanol Expansion in the United States*, another 2007 USDA publication, predicted that the “carryover” of corn (the corn on hand just before the next harvest, which is considered a good measure of the balance between supply and demand), will remain tight for another 10 years at 4–6% of annual consumption.

O’Neil isn’t so certain of U.S. corn-growing capacity: “If ethanol consumes eighteen to twenty percent of the corn crop, can we provide that? The answer is, ‘We think so, but nobody knows for sure,’ and the reason is that it depends on Mother Nature, because the single largest determinant of the crop is weather.”

## Long-Term Impact

In an attempt to plumb international impact of biofuel production, IFPRI director general Joachim von Braun projected in the February 2008 report *Food Prices, Biofuels and Climate Change* that worldwide calorie consumption would fall by 2% in most regions by 2020 if the trend toward biofuels is “moderate.” But a “drastic” biofuel expansion would reduce calorie consumption by more than 8% in Latin America and sub-Saharan Africa—a devastating reduction for someone who is already hungry.

Questions about biofuels highlight the complicated structure of agricultural markets: prices reflect supply and demand, farmer decisions, weather, crop diseases, distance to market, and the price of alternative crops. If demand raises the price of corn, farmers will plant more corn, raising the yield and reducing the price. But if that corn is planted on land formerly devoted to soybeans, the price of soybeans and cooking oil also may rise as the effects echo through the food markets.

Markets can stimulate production, notes O’Neil. “In order to encourage expansion of food and feed grains in the world,” he says, “we must have better prices for agricultural products to motivate farmers to invest in land and inputs.” He adds, “Even without ethanol and biodiesel, we need to motivate farmers around the world to expand production, and this can only be done through price incentives.”

Now that food crops can be converted into fuels, a new factor must be considered—the link between the price of food and the price of petroleum. As petroleum fuels get more expensive, biofuels become more profitable; therefore, biofuel producers can afford to pay more for their feedstock.

According to Brown, this new relationship puts hungry people in direct competition with empty gas tanks. “Historically the food and energy economies have been largely separate, but now with the construction of so many fuel ethanol distilleries, they are merging,” he says. “If the food value of grain is less than its fuel value, the market will move the grain into the energy economy. Thus, as the price of oil rises, the price of grain follows it upward.”

And that could mean more hunger for more people, says Runge, who participated in the FAO’s High-Level Conference on World Food Security and the Challenges of Climate Change and Bioenergy in Rome in February 2008. Most of the 82 countries that import food are also net oil importers, Runge says, so this competition between food and fuel harms people who are already “in a world of hurt.”

## Figures and Tables

**Figure:**
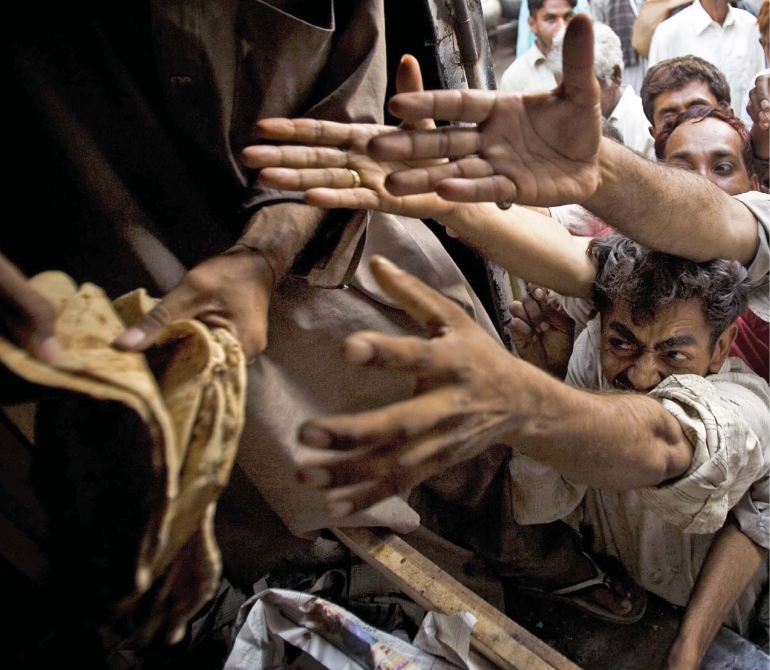
Food distribution in Lahore, Pakistan, 4 May 2008. Rising food prices not only have placed millions more people at risk for going hungry but also may impede how much aid agencies such as the World Food Programme can offer.

